# SbWRKY62 Coordinates With SbERF096 to Activate Pterostilbene Biosynthesis and Targets a Xylanase Inhibitor Protein for Anthracnose Resistance in Sorghum

**DOI:** 10.1111/pce.70833

**Published:** 2026-08-19

**Authors:** Yajing Li, Bofu Deng, Yiming Gan, Nan Lin, Dan Zhou, Clive Lo

**Affiliations:** ^1^ School of Biological Sciences The University of Hong Kong Hong Kong China; ^2^ College of Life and Environmental Science Wenzhou University Wenzhou China

**Keywords:** anthracnose, *Colletotrichum sublineola*, pterostilbene, SbERF096, SbWRKY62, SbXIP1, sorghum bicolour, transcriptional regulation

## Abstract

Sorghum anthracnose caused by *Colletotrichum sublineola* (*C. sublineola*) is a destructive disease leading to severe crop damages. Pterostilbene is a phytoalexin that contributes to defence responses in sorghum, but the transcriptional regulation of its biosynthesis remained poorly understood. Here, we characterised two transcription factors, SbERF096 and SbWRKY62, that are induced upon *C. sublineola* infection, with significantly higher expression levels in the resistant cultivar SC748‐5 than the susceptible cultivar BTx623. SbERF096 directly activates *SbSTS1*, which encodes stilbene synthase, via binding to GCC box, while SbWRKY62 directly activates *SbSOMT*, which encodes stilbene *O*‐methyltransferase (SOMT), via binding to W‐box motifs, marking the first transcription factor known to regulate a SOMT gene. Transient co‐expression of SbERF096 and SbWRKY62 promoted *SbSTS1* and *SbSOMT* expressions from their native promoters, respectively, resulting in elevated pterostilbene accumulation in *Nicotiana benthamiana* which does not produce stilbenes inherently. Furthermore, SbWRKY62 directly targets *SbXIP1* which is predicted to encode a xylanase inhibitor protein, a cell wall defence‐related protein that may reinforce physical barriers. These findings suggested that SbWRKY62 could confer anthracnose resistance with dual defence pathways to induce phytoalexin with SbERF096 and activate XIP production. Our work provides new molecular targets for breeding sorghum with enhanced anthracnose resistance.

## Summary statement

In sorghum (*Sorghum bicolour*), SbERF096 and SbWRKY62 activate pathogen‐inducible pterostilbene biosynthesis by regulating *SbSTS1* and *SbSOMT*, while SbWRKY62 also targets a xylanase inhibitor gene linked to anthracnose defence.

## 1 Introduction

Phytoalexins are inducible antimicrobial compounds that contribute to plant defence by restricting pathogen growth and are often associated with disease resistance (Hammerschmidt 1999). Stilbenes are a small group of plant polyphenols generally function as phytoalexins, offering protection against pathogen attacks (Jeandet et al. 2014) and abiotic stresses such as UV radiation (Adrian et al. 2000). In addition to their roles in plant defence, stilbenes are well‐recognised as promising health‐promoting nutraceuticals for humans due to their anti‐oxidation, anti‐tumour, anti‐obesity, anti‐diabetic, and cardioprotective properties (Chong et al. 2009; Reinisalo et al. 2015).

The general phenylpropanoid pathway (PPP) supplies *p*‐coumaroyl CoA as a common substrate for biosynthesis of different polyphenolic metabolites. Accordingly, phenylalanine is converted into *p*‐coumaroyl CoA by sequential actions of phenylalanine ammonia‐lyase, cinnamate‐4‐hydroxylase and 4‐coumarate: CoA ligase (Herrmann and Weaver (1999)). In land plants, chalcone synthase channels p‐coumaroyl CoA to flavonoids after three successive condensations with malonyl‐CoA (Winkel‐Shirley (2001). In stilbene‐accumulating plants, stilbene synthase (STS) utilises the same substrates to initiate the biosynthesis of stilbenes (Chong et al. 2009; Tropf et al. 1994). Despite the ubiquitous occurrences of PPP and flavonoid biosynthesis in plants, stilbenes were only isolated from a small number of diverse species such as resveratrol, piceid and pterostilbene in grapevine (*Vitis vinifera*, dicot), resveratrol in peanut (*Arachis hypogaea*, dicot), pinosylvin and pinosylvin monomethylether in Scots pine (*Pinus sylvestris*, gymnosperm), resveratrol, piceid, and pterostilbene in sorghum (*Sorghum bicolour*, monocot) and piceatannol in sugarcane (*Saccharum* spp., monocot) (Langcake et al. 1979; Brinker and Seigler (1991); Chiron et al. 2000; Sobolev et al. 2006; Yu et al. 2005).

Sorghum (*Sorghum bicolour*) is the fifth‐ranking cereal after wheat, maize, rice and barley. As a C4 plant, it is cultivated worldwide for food, animal feed and biofuel production. In addition, sorghum harbours biosynthetic pathways of many evolutionarily unique specialised metabolites involved in the mitigation of biotic and abiotic stresses. For instance, sorghum produces dhurrin, a cyanogenic glycoside, in its vegetative tissues and seeds as a chemical defence against herbivores (Vetter (2000). A distinct class of pigmented flavonoids, the 3‐deoxyanthocyanidins, accumulate in sorghum grains as antioxidants (Awika et al. 2004), in addition to their iconic role as phytoalexins (Snyder and Nicholson 1990). Recently, we further illustrated that pterostilbene is a new phytoalexin with strong inhibitory effects on *Colletotrichum sublineola*, the causal agent of sorghum anthracnose (Lui et al. 2023). The reaction steps for pterostilbene biosynthesis in sorghum upon pathogen infection have also been elucidated (Lui et al. 2023). Pterostilbene is a 3,5‐bis‐*O*‐methylated derivative of resveratrol. Accordingly, SbSTS1 catalyses the production of resveratrol from *p*‐coumaroyl CoA and malonyl CoA. Afterwards, stilbene *O*‐methyltransferase (SbSOMT) catalyses sequential *O*‐methylation of resveratrol to pinostilbene and then pterostilbene. Phylogenetic analyses further illustrated that SbSOMT was recruited from a canonical caffeic acid *O*‐methyltransferase subsequent to speciation in the Saccharinae subtribe which includes sorghum and sugarcane (Lui et al. 2023). Hence, the SOMT activities and the resulting metabolite pterostilbene are unique to the *Sorghum* genus among Saccharinae grasses.

Despite the identification of two key enzymes for pathogen‐inducible pterostilbene biosynthesis in sorghum, knowledge about the transcriptional regulation of STS and SOMT genes remained limited in monocots. By contrast, a number of transcription factors modulating stilbene biosynthesis have been characterised in grapevine which harbours 47 STS genes including 12 pseudogenes (Orduña et al. 2022). VvMYB14 and VvMYB15 were initially identified as positive transcriptional regulators of STS genes (Höll et al. 2013). In addition, several WRKY factors function either alone or cooperatively with MYB14 and/or MYB15 in modulating STS gene expression (Yin et al. 2022; Jiang et al. 2019; Mu et al. 2023; Wang et al. 2020; Vannozzi et al. 2018). VvWRKY24 alone up‐regulates *VvSTS29* transcription whereas VvWRKY3 requires VvMYB14 as a co‐effector to activate *VvSTS29* transcription (Vannozzi et al. 2018). The WRKY53‐activated VvSTS32 and VvSTS41 transcriptions were further enhanced when WRKY53 interacts with MYB14 or MYB15 (Wang et al. 2020). Furthermore, the involvement of ERF transcription factors for STS gene regulation was reported in Chinese wild grape (*Vitis quinquangularis* accession'Danfeng‐2) recently (Yan et al. 2025). VqERF1B activates *VqNSTS2* transcription by direct binding to its promoter while VqERF062 also forms a complex with VqERF1B to further promote the gene expression (Yan et al. 2025). Meanwhile, there has been no reports on transcriptional regulation of SOMTs such as grapevine VvROMT (Schmidlin et al. 2008) or Scots pine PsPMT2 (Paasela et al. 2017).

In this study, we identified two new pathogen‐inducible transcription factors in sorghum, SbERF096 (Sb01g000352) and SbWRKY62 (Sb05g001200), which up‐regulate *SbSTS1* and *SbSOMT* by binding to GCC box and W‐box motifs, respectively, promoting the biosynthesis of pterostilbene *in planta*. Concurrently, SbWRKY62 activates the expression of *SbXIP1 (Sb05g027380)* predicted to encode an inhibitor of xylanase which digests plant cell wall, hence functioning as a dual regulator of distinct defence responses. This work provides new insights into transcriptional regulation of pterostilbene biosynthesis as well as resistance breeding in sorghum against anthracnose.

## 2 Results

### 2.1 Transactivation of Pathogen‐Induced TFs on the Promoters of *SbSTS1* and *SbSOMT*

To investigate the transcriptional regulation of stilbene biosynthesis in sorghum, the promoters of *SbSTS1* and *SbSOMT* were first analysed by PlantCARE and PlantPAN4.0. MYB, ERF, bZIP, HD‐ZIP, bHLH, zinc finger, NAC and WRKY binding sites are located in both promoters (Supporting Information Figure S1b). Meanwhile, a transcriptome dataset for sorghum (cv. BTx623) seedlings infected with *C*. *sublineola* (Wang et al. 2020) was analysed to retrieve differentially expressed genes (at least 2.0‐fold increase) encoding transcription factors belonging to these families (Supporting Information Figure S1c). A total of 35 genes from selected families (Supporting Information Figure S2) were analysed for their transactivation activities on the promoters of *SbSTS1* and *SbSOMT*. Results from dual luciferase (LUC) reporter (DLR) assays (Figure 1) using tobacco (*Nicotiana benthamiana*) leaves revealed that 3 MYBs, including SbJAMYB, SbM000480 and SbMYB2‐like, activated the *SbSOMT* promoter by approximately 4.0‐, 3.0‐, 9.0‐fold increases in LUC activities, respectively. In addition, SbWRKY6 and SbWRKY62 activated the promoter of *SbSTS1* (2.0‐fold) and *SbSOMT* (6.0‐fold), respectively. SbERF096 also activated the *SbSTS1* promoter by approximately 2.0‐fold. The stronger activation of the *SbSOMT* promoter in the DLR assay may reflect higher promoter responsiveness to the tested transcription factors, possibly due to differences in promoter architecture, including the number, position, and sequence context of *cis*‐elements. On the other hand, none of the selected zinc fingers, NACs, ERFs and bHLHs showed transactivation effects on either promoter.

**Figure 1:**
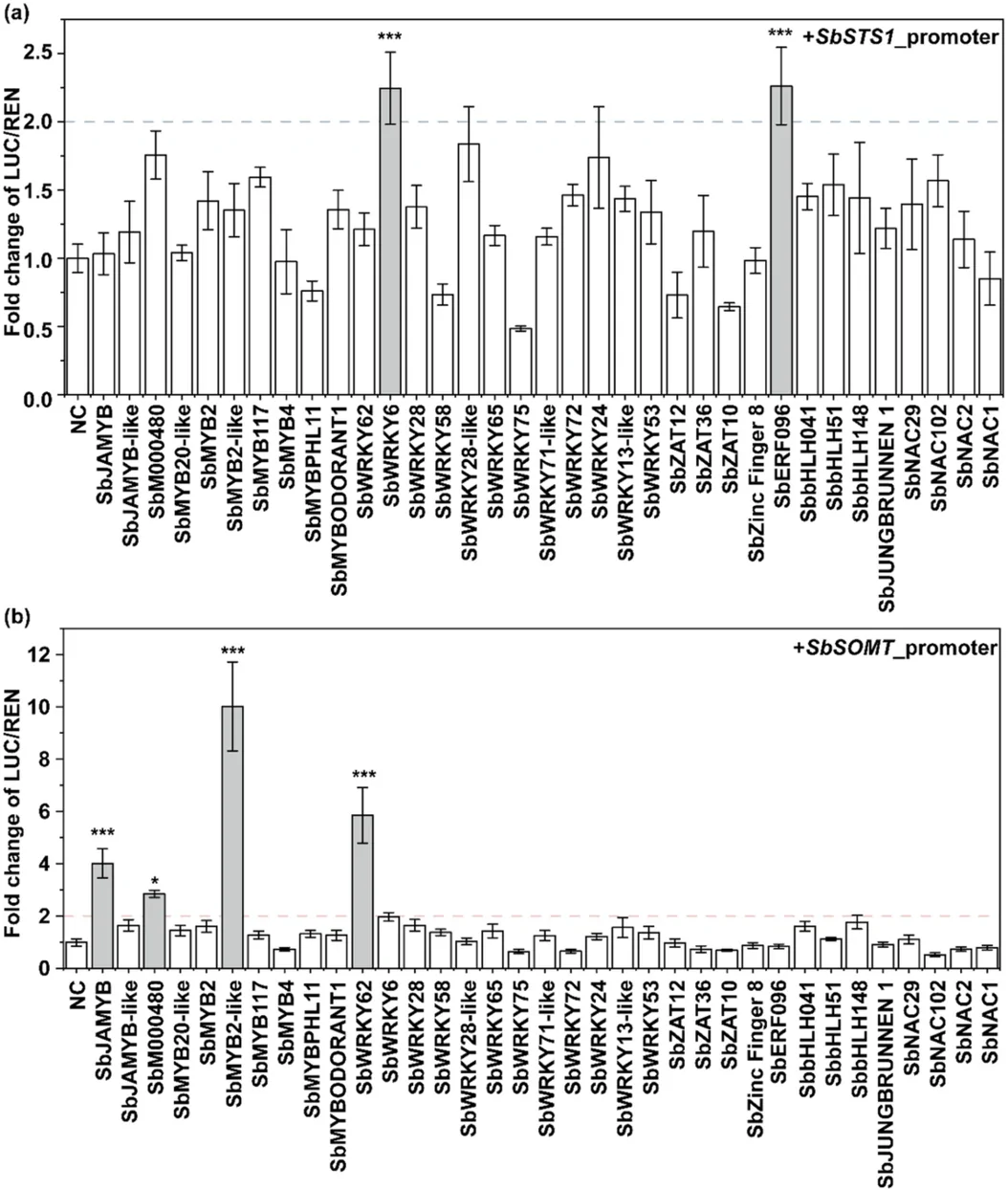
DLR assays for trans‐activation of selected pathogen‐inducible MYBs, WRKYs, Zinc Fingers, ERF, bHLHs, and NACs on the promoters of *SbSTS1* (a) and *SbSOMT* (b). Solid columns indicate TFs that promoted at least a 2.0 ‐fold change in LUC/REN ratio with negative control (NC). Values refer to means ± SE (*n* = 6). Statistical significance was determined using one‐way ANOVA followed by Dunnett's multiple comparison test. Asterisks indicate significant differences (*p < 0.05, ***p < 0.001).

### 2.2 Overexpression of Pathogen‐Induced TFs in Sorghum Protoplasts

The regulatory effects of SbJAMYB, Sb06g000480, SbMYB2‐like, SbWRKY6, SbWRKY62 and SbERF096 were subsequently investigated in sorghum protoplasts prepared from 18‐day‐old seedlings. Overexpression (OE) constructs for each gene under the control of 35S promoter were transfected individually into sorghum protoplasts, and gene expression was analysed by qRT‐PCR 16 h after transfection. Expression levels of *SbJAMYB, SbM000480, SbMYB2‐like, SbWRKY6, SbWRKY62* and *SbERF096* in the respective OE protoplasts were substantially higher than the control protoplasts. In *SbJAMYB* and *SbERF096* OE protoplasts, *SbSTS1* was significantly up‐regulated (Figure 2a,f), while *SbSOMT* was exclusively up‐regulated in SbWRKY62 OE protoplasts (Figure 2d). In contrast, OE of the other two MYBs (Sb06g000480, SbMYB2‐like) and SbWRKY6 did not have significant effects on the expression of either *SbSTS1* or *SbSOMT* (Figure 2b,c,e). Overall, these results suggested that SbERF096 and SbWRKY62 may participate in the regulation of *SbSTS1* and *SbSOMT* expression, respectively, in sorghum protoplasts.

**Figure 2:**
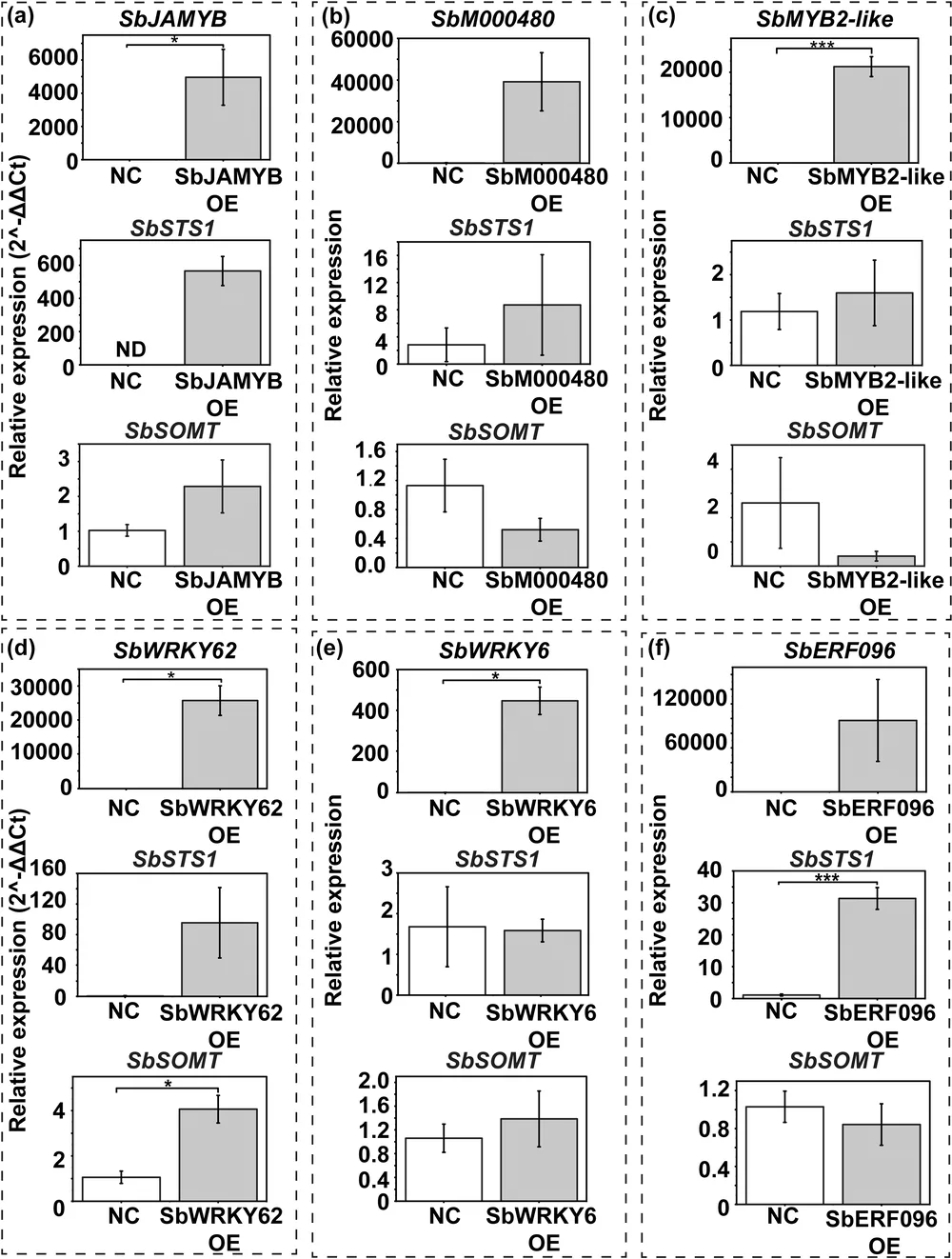
Gene expression in MYBs, WRKYs, and ERF overexpression sorghum protoplasts. qRT‐PCR analysis of *SbJAMYB*, *SbM000480*, *SbMYB2‐like*, *SbWRKY62, SbWRKY6, SbERF096* and *SbSTS1 and SbSOMT* expression in sorghum protoplasts over‐expressing *SbJAMYB* (a), *SbM000480* (b), *SbMYB2‐like* (c), *SbWRKY62* (d), *SbWRKY6* (e) or *SbERF096* (f). Relative expression levels were calculated using the 2^‐ΔΔCt method, with the empty‐vector control serving as the calibrator. Values refer to means ± SE (*n* = 3). NC, negative control; sorghum protoplasts transformed with the empty vector. ND, not detected. Statistical significance was determined using an unpaired two‐tailed Student's t‐test. Asterisks indicate significant differences (*p < 0.05, ***p < 0.001).

### 2.3 Activation of Pterostilbene Accumulation by SbERF096 and SbWRKY62 *in planta*

To investigate the potential roles of SbERF096 and SbWRKY62 in regulating SbSTS1‐ and SbSOMT‐mediated stilbene biosynthesis, *in planta* functional assays were performed via heterologous expression in tobacco, which does not possess an intrinsic stilbene biosynthetic pathway. *SbSTS1* under the control of its own promoter was transiently expressed in tobacco leaves, after which piceid (resveratrol *O*‐glucoside) was detected (Figure 3b). Apparently, SbSTS1 used the commonly available *p*‐coumaroyl CoA and malonyl CoA substrates to produce resveratrol which was glucosylated to piceid by endogenous UDP‐glucosyltransferases in tobacco. Moreover, co‐expression of SbERF096 under the control of the 35S promoter elevated piceid accumulation significantly compared to the empty SK control (Figure 3b). In contrast, a non‐activating ERF control (Sb01g040280) did not increase piceid accumulation relative to the SK control. These results support a role for SbERF096 in promoting SbSTS1‐mediated resveratrol production

**Figure 3:**
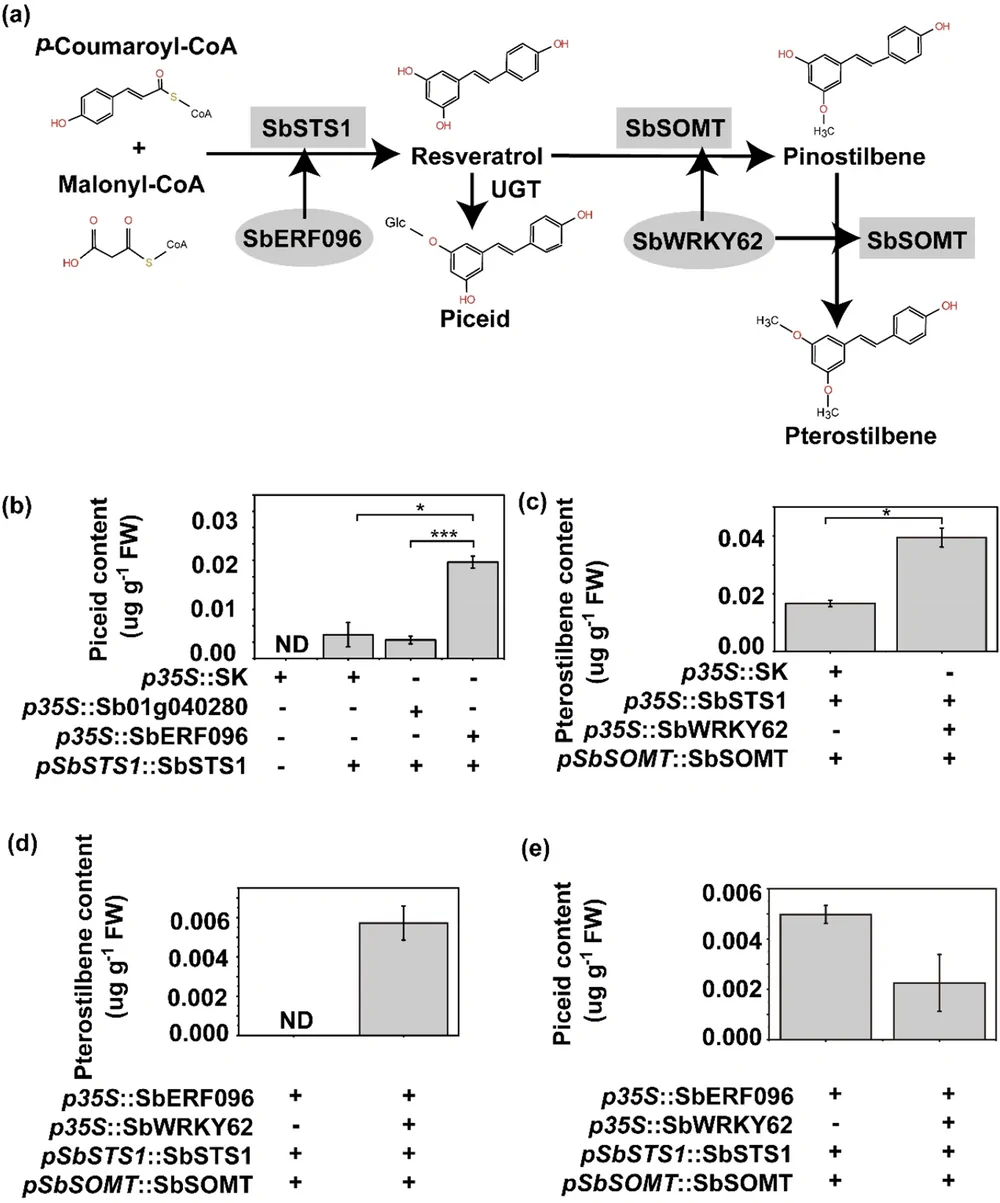
Metabolite analysis of tobacco leaves transiently over‐expressing SbERF096 and SbWRKY62. (a) Biosynthesis of pterostilbene sorghum. SbERF096 and SbWRKY62 up‐regulated *SbSTS1* and *SbSOMT*, respectively. (b) Accumulation of piceid in tobacco leaves transiently expressing p*SbSTS1*::SbSTS1 together with p*35S*::SbERF096, p*35S*::Sb01g040280, or the empty SK vector. Tobacco leaves infiltrated with the empty p*35S*::SK vector alone were used as the negative control. *Sb01g040280*, an AP2/ERF domain‐containing transcription factor that did not activate the *SbSTS1* promoter in the DLR assay, was included as a non‐activating ERF negative control. (c) Accumulation of pterostilbene in tobacco leaves co‐infiltrated with *p35S*::SbSTS1, *p35S*::SbWRKY62, and *pSbSOMT*::SbSOMT or *p35S*::SbSTS1, empty *p35S*::SK vector and *pSbSOMT*::SbSOMT. (d) Accumulation of pterostilbene in tobacco leaves co‐infiltrated with *p35S*::SbERF096, *p35S*::SbWRKY62, *pSbSTS1*::SbSTS1 and *pSbSOMT*::SbSOMT or *p35S*::SbERF096, *pSbSTS1*::SbSTS1, empty *p35S*::SK vector, and *pSbSOMT*::SbSOMT. (e) Accumulation of piceid in tobacco leaves co‐infiltrated with *p35S*::SbERF096, *p35S*::SbWRKY62, *pSbSTS1*::SbSTS1 and *pSbSOMT*::SbSOMT or *p35S*::SbERF096, *pSbSTS1*::SbSTS1, empty *p35S*::SK vector, and *pSbSOMT*::SbSOMT. Values are expressed as means ± SE (*n* = 3). ND, not detected. Statistical significance was determined using an unpaired two‐tailed Student's t‐test. Asterisks indicate significant differences (*p < 0.05, ***p < 0.001).

Subsequently, SbSTS1 was overexpressed under the 35S promoter in tobacco leaves to provide sufficient resveratrol as substrate for SbSOMT. As shown in Figure 3c, pterostilbene was detected when SbSOMT under the control of its own promoter was co‐expressed with 35S::SbSTS1 in tobacco leaves (Figure 3c). Moreover, co‐expression of SbWRKY62 under the 35S promoter further enhanced pterostilbene production significantly (Figure 3c), supporting a role for SbWRKY62 in promoting SbSOMT‐mediated pterostilbene production.

Finally, we co‐expressed SbSTS1 and SbSOMT (each with its own promoter) with SbERF096 and SbWRKY62 (each with 35S promoter) in tobacco leaves, resulting in accumulation of pterostilbene which is not detected in the negative control (Figure 3d). Piceid was also detected in SbSTS1‐containing samples. However, its accumulation did not further increase when p*35S*::SbWRKY62 and p*SbSOMT*::SbSOMT were included, consistent with conversion of resveratrol toward pterostilbene production. Taken together, our results conclusively established that SbERF096 and SbWRKY62 transcriptionally activate *SbSTS1* and *SbSOMT*, respectively, leading to biosynthesis of pterostilbene *in planta*.

### 2.4 Expression Analysis of SbERF096 and SbWRKY62 in Infected Sorghum

As defence metabolites, stilbenes are often synthesised under biotic and abiotic stresses. Gene expression analysis was performed using two sorghum cultivars, BTx623 and SC748‑5, which are susceptible and resistant to sorghum anthracnose, respectively. Results showed that *SbERF096* and *SbWRKY62* were up‐regulated from 24 h to 72 h post‐infection in both lines, with expression levels significantly higher in SC748‐5 than BTx623 (Supporting Information Figure S3a,b), consistent with the expression patterns previously observed for *SbSTS1* and *SbSOMT* (Lui et al. 2023).

### 2.5 Phylogenetic Analysis of SbERF096 and SbWRKY62

Phylogenetic analysis of SbERF096 with its homologues from monocot and dicot species categorised the ERF proteins into five distinct clades. Dicot homologues are clustered with several non‐grass monocot homologues in Clade I. On the other hand, there are two large clades of grass homologues. SbERF096 is clustered in Clade IV with ERF proteins from closely‐related grasses in Andropogoneae tribe, including AgERF1 (*Andropogon gerardi*), MsERF1 (*Miscanthus sinensis*), SsERF1 (*Saccharum officinarum x spontaneum*), and ZmERF1 (*Zea mays*) (Supporting Information Figure S4). Meanwhile, SbWRKY62 clusters with WRKY homologues from closely‐related Andropogoneae species, including MisinT027900 (*Miscanthus sinensis*), AndgeH1.05BG011200.1 (*Andropogon gerardi*), 10Ag014700.1 (*Saccharum officinarum × spontaneum*), and ZmWRKY70‐like (*Zea mays*), within Clade II (Supporting Information Figure S5). Taken together, both SbERF096 and SbWRKY62 are highly conserved with their respective homologues from the closely‐related Andropogoneae grasses.

### 2.6 Interaction of SbERF096 and *SbSTS1* Promoter

The 1500‐bp promoter region of *SbSTS1* was divided into three 500‐bp segments (S1, S2 and S3) and individually cloned into the pLacZi vector (Figure 4a). Only yeast colonies containing S3 exhibited blue coloration, indicating that the direct binding of SbERF096 occurred within S3 (Figure 4b). Previously, ERFs recognised GCC box, DRE, and RAA motifs (Yan et al. 2025) but only GCC box (GCCGAC) and RAA motif (CAACA) were found within S3 (Figure 4a). Chromatin immunoprecipitation (ChIP)‐qPCR showed a 4.0‐fold enrichment of in vivo binding of Flag‐tagged SbERF096 to GCC box‐containing fragment (Figure 4c) but no significant enrichment for the RAA motif‐containing fragment. Electrophoretic mobility shift assay (EMSA) confirmed in vitro binding of SbERF096 to the biotin‐modified GCC probe (Figure 4a,d), while increasing concentrations of cold probe of GCC box reduced the binding signals.

**Figure 4:**
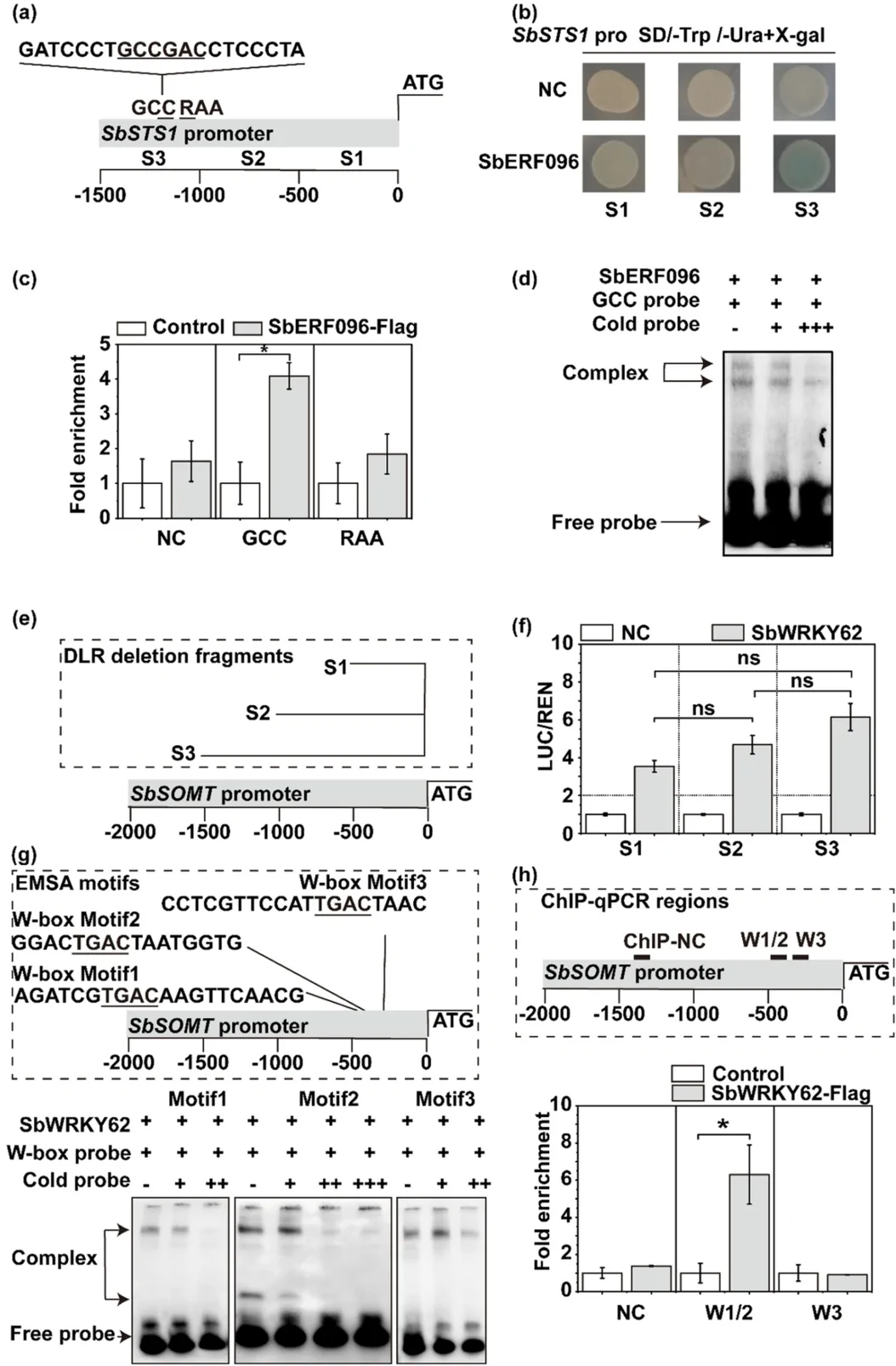
SbERF096 trans‐activates *SbSTS1* by directly binding to GCC motif in the promoter and SbWRKY62 trans‐activates *SbSOMT* by directly binding to W‐boxes. (a) Schematic diagram of 3 segments (S1 to S3) of the promoter, locations of GCC‐motif and RAA‐motif in ChIP‐qPCR and sequence of GCC motif. (b)Y1H (blue colony) suggested SbERF096 bound to S3, but not S1 or S2 in the promoter of *SbSTS1*. (c) ChIP‐qPCR demonstrated that SbERF096 could bind directly to GCC motif, but not RAA motif in vivo. Fold enrichment values were normalised to the input, which was set to 1. Values refer to means ± SE (*n* = 3). Asterisks indicate significant differences at *p < 0.05. (d) EMSA demonstrated SbERF096 could bind to GCC‐motif (biotinylated probe) *in vitro*. SbERF096 was purified as a fusion protein with an MBP tag. Cold probes were not biotinylated. For competition assays, +, and +++ indicate 50‐ and 500‐fold molar excesses of unlabelled competitor probe, respectively. (e) In the *SbSOMT* promoter schematic, S1–S3 indicate promoter deletion fragments used for the DLR assay. (f) DLR assays demonstrated 4‐, 5‐, 6‐fold trans‐activation of S1, S2 and S3 promoter segments, respectively, by SbWRKY62. Negative controls (NC) were standardised to 1. Values refer to means ± SE (*n* = 6). (g) EMSA suggested that SbWRKY62 could bind to W‐boxes of 3 motifs (biotinylated probes) in vitro. SbWRKY62 was purified as a fusion protein with MBP tag. Cold probes were not biotinylated. ‘+’ or ‘–’ indicates presence or absence, respectively. For competition assays, +, ++ and +++ indicate 50‐, 250‐, and 500‐fold molar excesses of unlabelled competitor probe, respectively. (h) ChIP‐qPCR demonstrated that SbWRKY62 could bind to motif1 and motif2, but not motif3 in vivo. W1/2 indicates the ChIP‐qPCR region containing W‐box Motif1 and W‐box Motif2, W3 indicates the region containing W‐box Motif3, and ChIP‐NC indicates a promoter region without a W‐box motif used as the negative control. Fold enrichments of Input were standardised to 1. Values refer to means ± SE (*n* = 3). Statistical significance was determined using an unpaired two‐tailed Student's t‐test. Asterisks indicate significant differences (*p < 0.05). [Color figure can be viewed at wileyonlinelibrary.com]

### 2.7 Interaction of SbWRKY62 and *SbSOMT* Promoter

A series of 5’‐deletions of the *SbSOMT* promoter (500, 1000 and 1500 bp upstream of the start codon) was generated for cloning into the LUC reporter construct (Figure 4e). All three promoter segments were activated by SbWRKY62, yielding around 4.0‐, 5.0‐ and 6.0‐fold increases in LUC activities, respectively (Figure 4f). Since the 500 bp segment (S1) alone was activated with no significant differences with S2 or S3, the key *cis*‐elements for SbWRKY62 binding are likely located within this region (Figure 4f). Consistently, we identified three W‐box motifs with the core TGAC sequence known to be recognised by WRKYs (Yamasaki et al. 2008). EMSA further confirmed SbWRKY62 binding to all three biotin‐labelled probes (W1, W2 and W3) for these motifs with signals substantially reduced by cold probes (Figure 4e,g). ChIP‐qPCR was employed to assess the in vivo interaction between SbWRKY62 and *SbSOMT* promoter. The flag‐tagged SbWRKY62 was enriched in the S1 segment containing both Motif 1 and Motif 2 (Figure 4e,h). Taken together, these results suggested that W‐box motif 1 and W‐box motif 2 cooperatively contributed to the functional interaction with SbWRKY62.

### 2.8 Identification of Additional Target Genes of SbWRKY62 by DNA Affinity Purification Sequencing (DAP‐seq)

As a newly‐identified WRKY unique in the grass family, SbWRKY62 directly activates *SbSOMT* expression during pathogen‐inducible pterostilbene production as illustrated above. Subsequently, DAP‐seq was performed to discover additional genes under direct regulation by SbWRKY62 during defence responses. As shown in Figure 5a, DAP‐seq peaks were enriched in regions flanking transcription start sites, spanning 2 kb upstream and downstream. Most of them were distributed on intergenic regions (63.5%), followed by promoter regions (12.2%) (Figure 5b). The top 3 significantly enriched motifs (1–3) for SbWRKY62 binding to promoter regions are shown in Figure 5c, with motif 1 having the highest frequency (19.57% of total). Both motif 1 and motif 3 contain the typical core‐binding element (5’‐TTGAC‐3’) for WRKYs.

**Figure 5:**
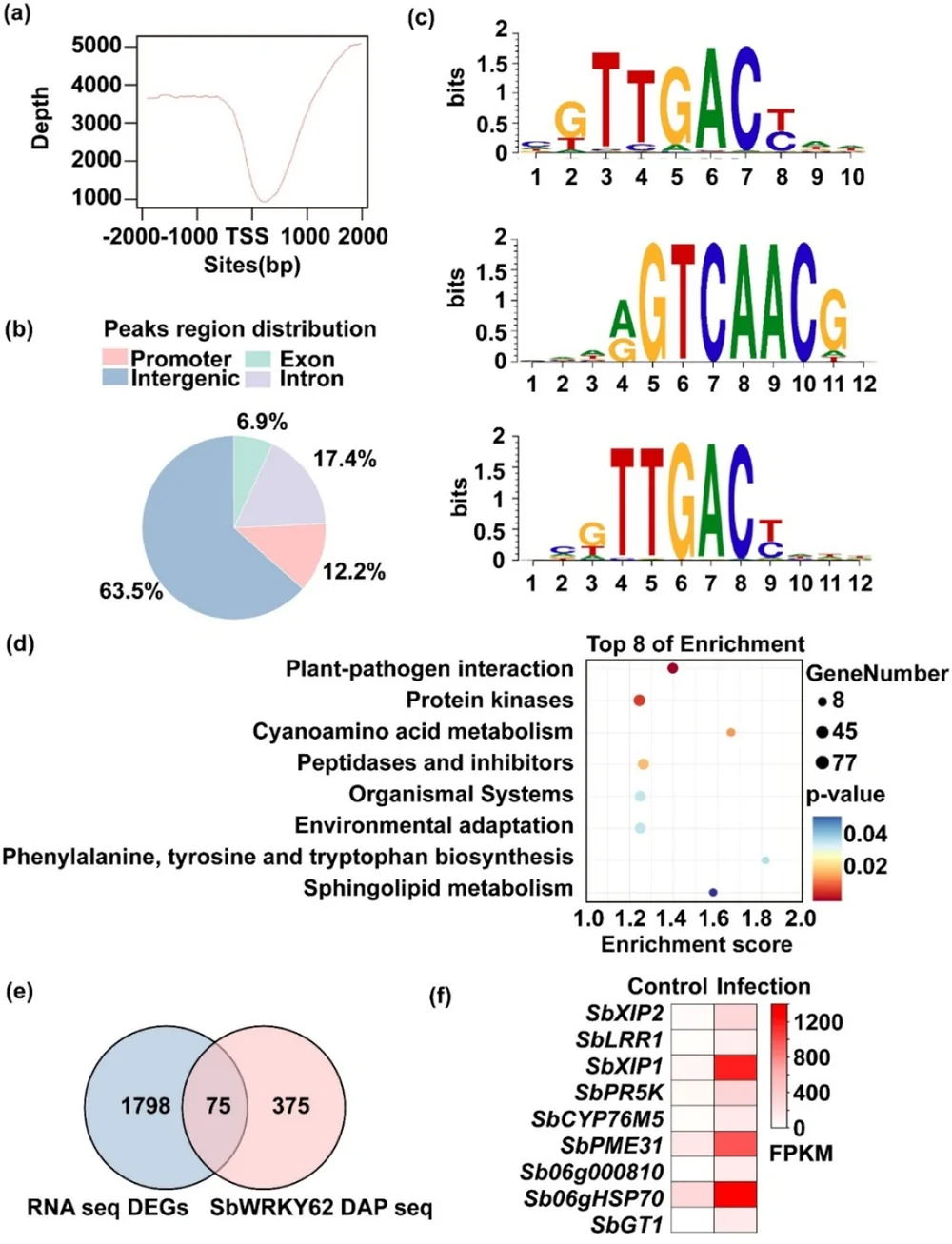
Combinatory analysis of DAP‐seq and RNA‐seq datasets identified potential target genes of SbWRKY62. (a) Distribution of DAP‐seq peaks (overlapped peaks in two replicates) based on distance to transcript start site (TSS). (b) Distribution of SbWRKY62‐binding peaks based on gene functional elements. (c) The top 3 conserved motifs enriched by SbWRKY62. (d) KEGG pathway enrichment analysis of SbWRKY62‐binding genes. Nominal *p*‐values are shown in the bubble plot, and multiple testing correction was performed using the Benjamini–Hochberg method. (e) Venn diagram of intersection DEGs by DAP‐seq and RNA‐seq (Accession: PRJNA525011). Fold change ≥ 2 as the threshold in RNA‐seq, and Fold enrichment ≥ 4 as the threshold in DAP‐seq. (f) Gene expression pattern of 9 candidates identified from RNA‐seq and DAP‐seq. [Color figure can be viewed at wileyonlinelibrary.com]

Two independent DAP‐seq replicates revealed 12 476 overlapping target genes in promoter regions. KEGG enrichment analysis of these genes (with 4555 showing a more than 4.0‐fold enrichment) highlighted plant‐pathogen interaction, biosynthesis of secondary metabolites, and environmental adaptation as the most significantly enriched pathways (Figure 5d). A combined analysis of infected sorghum RNA‐seq (Wang et al. 2020) and DAP‐seq datasets was then conducted to identify new candidates for SbWRKY62‐regulated defence‐related genes. After intersecting 1873 differentially expressed genes (2.0‐fold increase) with 450 DAP‐seq target genes with a high enrichment score (15‐fold), a total of 75 high‐confidence candidate genes were identified (Figure 5e). From this pool, 9 candidates were shortlisted based on KEGG pathway annotation and motif prediction. They were classified into three groups: 6 in Class I (Plant‐pathogen interaction), 2 in Class II (Environmental adaptation), and 1 in Class III (Secondary metabolites) (Figure 5f). The predicted binding regions of these genes are shown in Figure S6.

Transactivation activities of SbWRKY62 on the candidate gene promoters were subsequently assessed by DLR assays. The previously validated p*35S*::SbWRKY62–p*SbSOMT*::LUC combination was included as an internal positive control and showed clear promoter activation, confirming the validity of the DLR assay. Their promoter sequences were cloned into LUC reporter vectors with SbWRKY62 serving as the effector. Resulted showed that SbWRKY62 specifically trans‐activated the promoter of *SbXIP1* (*Xylanase Inhibitor Protein1*), resulting in an approximately 2.0‐fold increase in LUC activity (Figure 6a). SbWRKY62 was over‐expressed in sorghum protoplasts and the transcript levels of *SbXIP1* were significantly elevated compared to the control (Figure 6b). Although *SbCYP76M5* expression was induced by SbWRKY62 overexpression in sorghum protoplasts, activation of the *SbCYP76M5* promoter by SbWRKY62 was relatively weak in the DLR assay. Therefore, we focused further analysis on *SbXIP1*, while considering *SbCYP76M5* as a possible indirectly regulated SbWRKY62‐responsive gene. XIPs are recognised as defence components in monocots (Zhan et al. 2017; Gao et al. 2020; Juge et al. 2004; Igawa et al. 2005; Payan et al. 2004) by inhibiting microbial xylanase activity which would otherwise degrade plant cell walls. There are six XIPs in sorghum (Phytozome 14) and 4 of them were pathogen‐induced according to FPKM values in RNA‐seq of infected BTx623 (Supporting Information Figure S7a). Phylogenetic analysis revealed that SbXIP1 is closely related to another sorghum XIP and wheat (*Triticum aestivum*) XIP1 (Supporting Information Figure S7b). Possible direct binding sites on the *SbXIP1* promoter were then analysed using the DAP‐seq data. Three potential binding motifs (1–3) were identified within the peak region (Figure 6c). Subsequent EMSA and ChIP‐qPCR assays confirmed that SbWRKY62 specifically bound to motif 2, but not to motif 1 or motif 3, both *in vitro* and *in vivo*, respectively (Figure 6d,e). Additionally, the expression level of *SbXIP1* in SC748‐5 was considerably higher than that in BTx623 at 72 h after infection (Supporting Information Figure S7c), implicating that SbXIP1 is also under the regulation of SbWRKY62 during defence response against *C*. *sublineola*.

**Figure 6:**
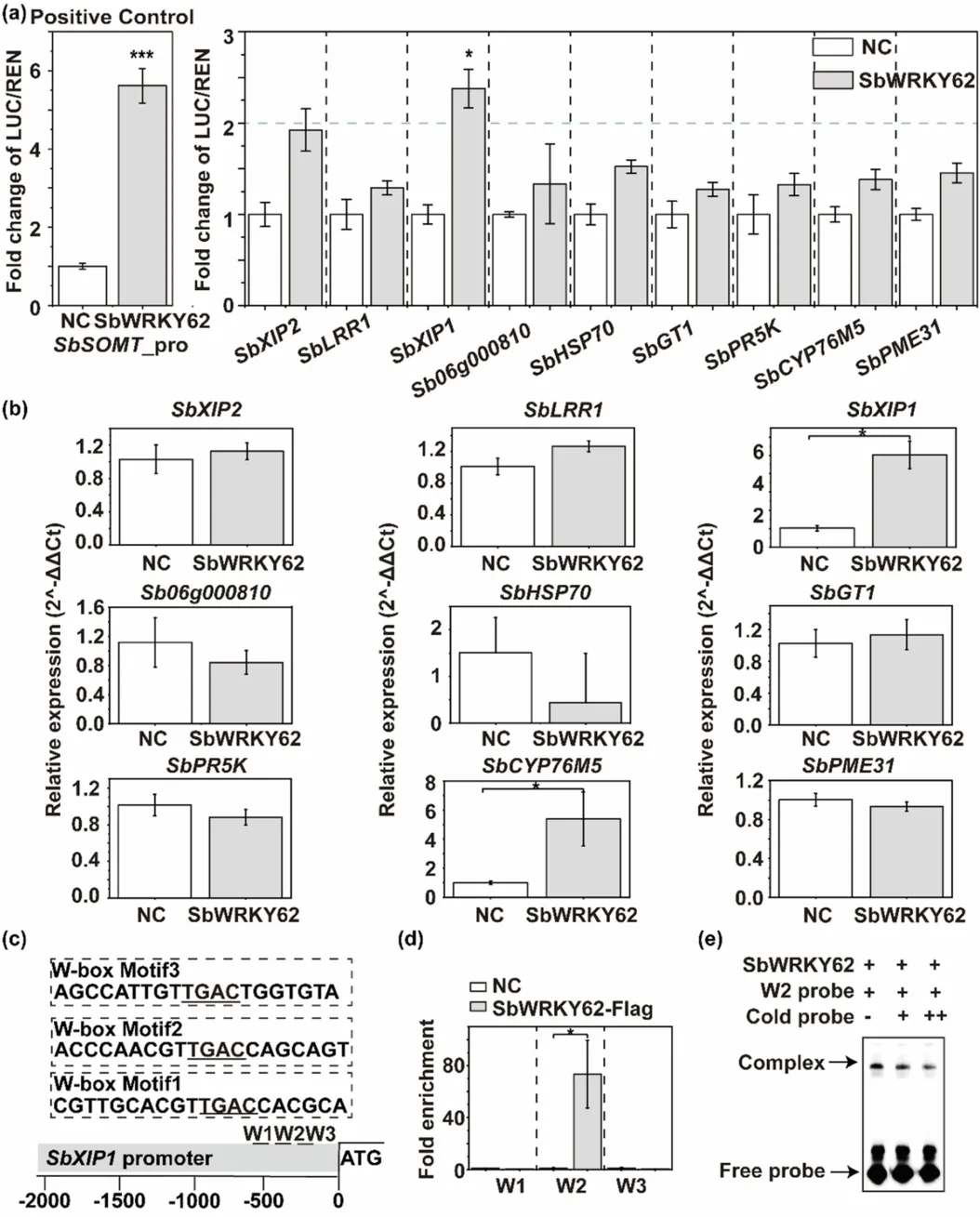
Regulation of *SbXIP1* by SbWRKY62. (a) DLR assays for transactivation of nine promoter candidates by SbWRKY62. The p*35S*::SbWRKY62 and p*SbSOMT*::LUC combination, which was previously validated to activate the *SbSOMT* promoter, was included as an internal positive control for the DLR assay. Values refer to means ± SE (*n* = 6). Statistical significance was determined using an unpaired two‐tailed Student's t‐test. Asterisks indicate significant differences (*p < 0.05, ***p < 0.001). (b) qRT‐PCR gene expression analysis of 9 candidate genes in SbWRKY62 over‐expressed sorghum protoplasts. Values refer to means ± SE (*n* = 3). Statistical significance was determined using an unpaired two‐tailed Student's t‐test. Asterisks indicate significant differences (*p < 0.05). (c) Schematic diagram of sequences of 3 W‐boxes in the promoter of *SbXIP1*. (d) ChIP‐qPCR demonstrated that SbWRKY62 could bind to W2 in vivo. Fold enrichments of Input were standardised to 1. Values refer to means ± SE (*n* = 3). Statistical significance was determined using an unpaired two‐tailed Student's t‐test. Asterisks indicate significant differences (*p < 0.05). (e) EMSA suggested SbWRKY62 could bind to W2 (biotinylated probe) in vitro. SbWRKY62 was purified as a fusion protein with an MBP tag. Cold probes were not biotinylated. ‘+’ or ‘–’ indicates presence or absence, respectively. For competition assays, + and ++ indicate 50‐, and 250 ‐fold molar excesses of unlabelled competitor probe, respectively.

## 3 Discussion

The ERF transcription factor family plays important roles in regulating the biosynthesis of secondary metabolites under both biotic and abiotic stresses (Chen et al. 2026). For example, the jasmonate‐responsive AaERF1 and AaERF2 in *Artemisia annua* are two positive regulators of artemisinin biosynthesis (Yu et al. 2012). In apple, MdERF1B activates the expression of *MdERF1*, *MdERF3* and *MdACO1* for ethylene biosynthesis as well as the expression of *MdMYB1*, *MYB9* and *MYB13* to accumulate anthocyanins and proanthocyanins (Zhang et al. 2018). In *Morella rubra* leaves, the repressor MrERF4 and the activator MrERF34 synergistically regulate high flavonol accumulation upon UV‐B irradiation (Li et al. 2025). In this study, our results support that the pathogen‐inducible SbERF096 from sorghum functions as a transcriptional activator of SbSTS1 which catalyses the first committed step in stilbene biosynthesis to produce resveratrol.

SbERF096 belongs to the ERF subfamily of the AP2/ERF transcription factor family, as it contains a single AP2/ERF domain with conserved alanine at position 14 (A14) and aspartic acid at position 19 (D19) (Sakuma et al. 2002). Phylogenetic analysis placed SbERF096 within a grass‐specific clade, distinct from its homologues in dicots and non‐grass monocots (Supporting Information Figure S4). In grapevines, several ERF transcription factors, including VqERF114, VqERF1B, and VqERF062, are involved in the regulation of STS gene expression (Yan et al. 2025). Particularly, VqERF062, which is phylogenetically unrelated to SbERF096, activates *VqNSTS2* transcription by binding to the DRE and RAA motifs in its promoter. On the other hand, VqERF1B, which is remotely related to SbERF096 with 35.1% sequence identity, functions as an indirect regulator by forming a VqERF1B‐VqERF062 complex to further enhance *VqNSTS2* transcription (Yan et al. 2025). It remains to be investigated whether SbERF096 also interacts with another ERF protein to further modulate *SbSTS1* transcription in sorghum. Nonetheless, SbERF096 apparently represents a grass‐specific ERF independently recruited for regulation of *SbSTS1* expression in sorghum through direct binding to the GCC motif within the promoter region which also contains the RAA motif but not the DRE motif (Figure 4a–d).

The plant‐specific WRKY transcription factors, characterised by the conserved WRKYGQK motif and a zinc finger domain, are often involved in plant immune responses (Wang et al. 2026). For example, OsWRKY67, when phosphorylated by OsMPK6, directly activates the expression of *OsNOMT* encoding naringenin *O*‐methyltransferase, leading to elevated production of sakuranetin which enhanced resistance in rice against false smut disease (Ma et al. 2025). In this study, our results support that the pathogen‐inducible SbWRKY62 from sorghum functions as a transcriptional activator of SbSOMT which catalysers sequential *O*‐methylation of resveratrol to produce pterostilbene.

SbWRKY62 belongs to group III WRKY family, distinguishing it from VqWRKY24 (group I) (Vannozzi et al. 2018), VqWRKY53 (group II‐c) (Wang et al. 2020), and VqWRKY8 (group II‐c) (Jiang et al. 2019; Mu et al. 2023) which were previously reported to regulate the expression of STS genes leading to resveratrol accumulation in grapevine. On the other hand, SbWRKY62 is a highly grass‐specific WRKY with no phylogenetically related homologues from other taxonomic groups. In fact, SbWRKY62 is highly conserved with WRKYs from Andropogoneae grasses including sugarcane, maize, *Miscanthus* and *Andropogon*. However, since SbSOMT orthologs are absent in these grasses, SbWRKY62 most probably acquired a unique regulatory role on *SbSOMT* gene expression in sorghum during speciation in the Andropogoneae tribe. Meanwhile, the three reported stilbene OMTs, including VvROMT (Schmidlin et al. 2008), PsPMT2 (Paasela et al. 2017) and SbSOMT (Lui et al. 2023), are phylogenetically distinct enzymes sharing less than 35% sequence identity with each other. To date, transcription factors modulating *VvROMT* expression in grapevine and *PsPMT2* expression in Scots pine remains unexplored. The underlying regulatory modules were likely recruited independently after the host plants had evolved the respective enzymes convergently to accept stilbenes as substrates for *O*‐methylation.

Sorghum anthracnose caused by *C*. *sublineola* is a highly destructive disease which causes damages in leaves, stems, panicles and grains, resulting in severe yield losses over 80% under severe epidemic conditions (Cota et al. 2017; Prom et al. 2022). Currently, the use of resistant germplasm remains the most efficacious and sustainable disease management strategy (Stutts and Vermerris (2020). SC748‐5 is a cultivar renowned for its strong resistance against sorghum anthracnose (Burrell et al. 2015). In this study, our results provide new insights into a potential molecular defence mechanism associated with anthracnose resistance in sorghum (Figure 7). Both *SbERF096* and *SbWRKY62* exhibited stronger expression levels in SC748‐5 than the susceptible cultivar BTx623 following *C*. *sublineola* inoculation. Consequently, their direct transcriptional activation of target genes encoding SbSTS1 and SbSOMT, respectively, would contribute to the elevated accumulation of pterostilbene which is highly inhibitory to spore germination and mycelial growth of the anthracnose pathogen (Lui et al. 2023). A suite of phytoalexins comprising 3‐deoxyanthocyanidins (Lo et al. 1999), flavones (Du et al. 2010), and pterostilbene (Lui et al. 2023), which are produced rapidly and abundantly in SC748‐5 after initial exposure to *C*. *sublineola*, is expected to act synergistically to interrupt the initial infection process. Nevertheless, stable genetic validation using overexpression lines or genome‐edited mutants will be required to directly determine the roles of SbERF096 and SbWRKY62 in sorghum anthracnose resistance. In addition, promoter motif editing or functional analysis of DNA‐binding‐defective transcription factor variants will be needed to determine whether these binding motifs or domains are genetically required for resistance to *C. sublineola*.

**Figure 7:**
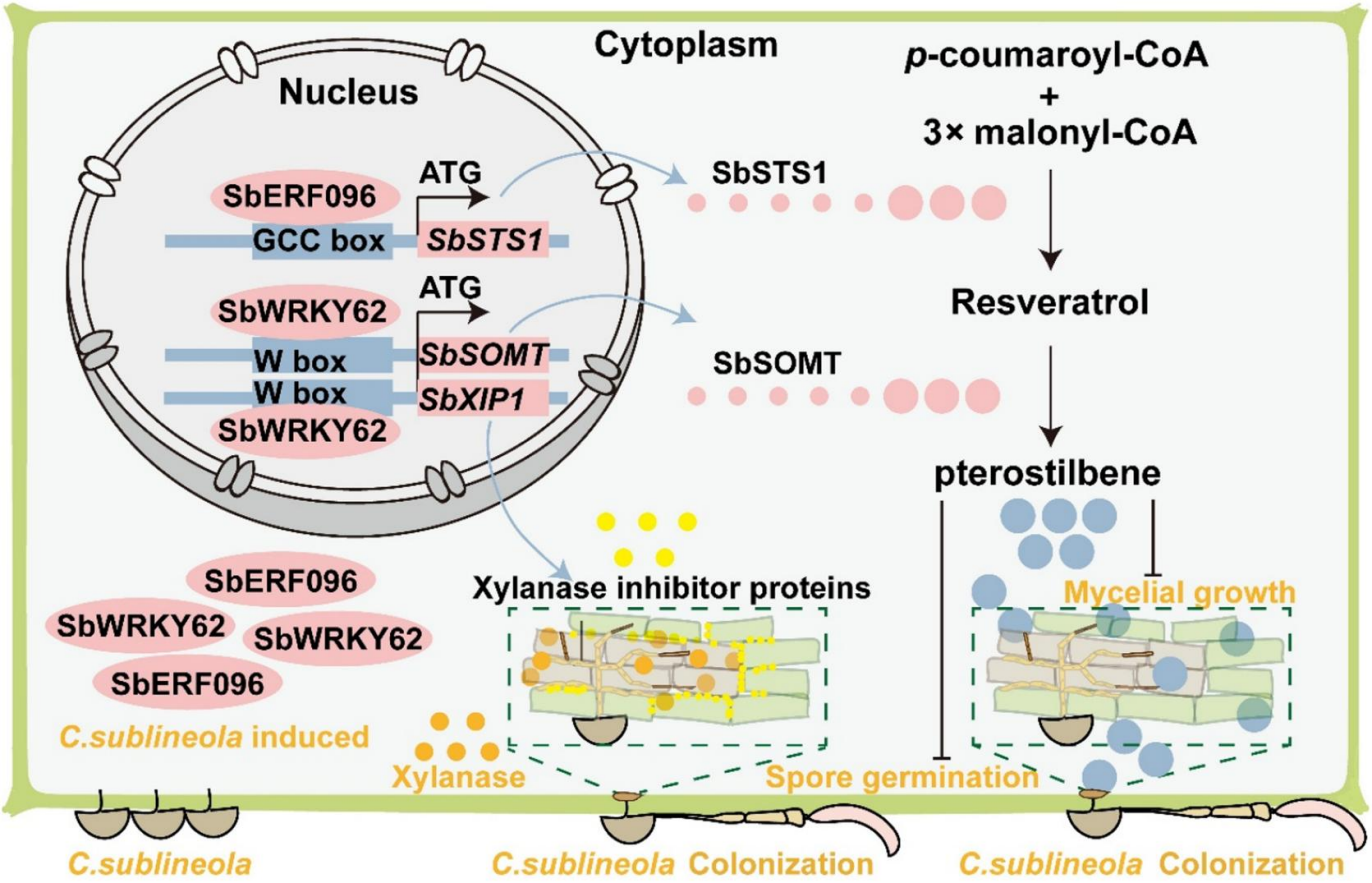
Proposed model of SbERF096 and SbWRKY62‐mediated plant defence against *Colletotrichum sublineola*. Pathogen‐induced SbERF096 and SbWRKY62 directly activate *SbSTS1* and *SbSOMT* via GCC box and W‐box, respectively, promoting pterostilbene accumulation to inhibit spore germination and mycelial growth of *C. sublineola*. Concurrently, SbWRKY62 upregulates *SbXIP1* by binding to the W‐box. SbXIP1 likely function to inhibit the activities of fungal xylanases, thus slowing down cell wall degradation and pathogen colonisation. Dead cells are shown in brown. [Color figure can be viewed at wileyonlinelibrary.com]

Our further characterisation of SbWRKY62 revealed an additional target gene *SbXIP1* which is predicted to encode a xylanase inhibitor protein (XIP). SbWRKY62 up‐regulates *SbXIP1* expression through direct binding to the W‐box motif within the promoter region. Consistent with the *SbWRKY62* expression pattern, *SbXIP1* expression is more strongly induced in SC748‐5 than BTx623 following *C*. *sublineola* inoculation. The fungus is a hemibiotrophic pathogen with an initial biotrophic phase followed by a destructive necrotrophic phase. During infection, fungal pathogens can secrete a cocktail of cell wall‐degrading enzymes, with such activities often becoming more prominent during the necrotrophic phase. These enzymes include xylanases which digest xylan, a predominant hemicellulose in monocot cell walls. Hence, SbXIP1 could function to slow down or restrict *C*. *sublineola* colonisation and symptom development by inhibition of xylanase. In fact, transgenic wheat lines expressing XIP‐I or TAXI‐III exhibit reduced *Fusarium graminearum* infection through direct inhibition of xylanase activity (Moscetti et al. 2013; Tundo et al. 2015). Similarly, overexpression of *RIXI* (rice xylanase inhibitor) enhanced disease resistance against *Magnaporthe oryzae* in rice (Hou et al. 2015). Furthermore, transcriptional regulation of XIPs has also been documented in rice. Overexpression of OsWRKY6 and OsWRKY46 in transgenic rice enhances disease resistance by promoted *RIXI* expression in transgenic rice with stronger disease resistance (Gao et al. 2020). Additionally, both OsbHLH59 and OsERF71 activate *OsXIP* expression by direct binding to a 562‐bp key sequence in the promoter region (Zhan et al. 2017). Meanwhile, many plant WRKY proteins are known to regulate multiple downstream genes directly. In rice, OsWRKY36 is a multifunctional transcriptional repressor of *OsPALs*, which is involved in phenylpropanoid pathway, as well as IPA and MOCs, which are related to spikelet and tiller numbers (Liu et al. 2025). In Arabidopsis, WRKY8 represses expression of the nlp20/nlp24 receptor gene *RLP23* while promoting that of the chitin receptor gene *CERK1*, revealing a 'kill two birds with one stone' strategy employed by plants to elicit robust broad‐spectrum immunity (Ren et al. 2024). In grapevine, VvWRKY18 directly activates the expressions of *VvSTS1* and *VvSTS2* by binding to their promoters, while simultaneously participating in the regulation of PR gene expression through its interaction with VvNPR1 (Wang et al. 2021). Similarly, our results suggest that SbWRKY62 may have a dual regulatory role to activate the expression of *SbSOMT* and *SbXIP1* by direct binding to the W‐box motifs within their promoter regions.

In conclusion, this work demonstrated that SbERF096 and SbWRKY62 are direct upstream regulators of pathogen‐inducible stilbene biosynthesis in sorghum. Particularly, it provides the first platform for elucidating the molecular regulation of pterostilbene biosynthesis in plants harbouring stilbene OMT enzymes. In addition, SbXIP1, which is co‐regulated by SbWRKY62, was revealed as a potential defence component against *C*. *sublineola* infection. Finally, our results provide new insights into the regulatory network of pterostilbene biosynthesis and offer candidate targets for future functional validation and molecular breeding of anthracnose resistance in sorghum.

## 4 Materials and Methods

### 4.1 Plant Materials and Fungal Inoculation

Two sorghum (*Sorghum bicolour* L.) cultivars BTx623 and SC748‐5 were used for fungal inoculation as described previously (Lo et al. 1996). The anthracnose pathogen *Colletotrichum sublineola* (isolate TX430BB) was cultured on 5% (wt/vol) oatmeal agar for 14 days prior to spore inoculation. Sorghum seedlings were germinated in moist paper rolls in the dark for 7 days. Etiolated seedlings were then sprayed with either a 2% bovine gelatin solution (control) or the same solution containing *C. sublineola* spores at a concentration of 1.0 × 10^6^ mL^−1^ (infection treatment). All plants were maintained under constant light at room temperature and 100% humidity.

### 4.2 Bioinformatics Analyses

The 2‐kb promoter sequences upstream of the transcription start site of *SbSTS1* and *SbSOMT* were analysed online using PlantCARE (https://bioinformatics.psb.ugent.be/webtools/plantcare/html/) and PlantPAN4.0 (https://plantpan.itps.ncku.edu.tw/plantpan4/index.html). Differentially expressed genes (DEGs) were identified from published transcriptome datasets (BTx623: PRJNA525011; Sc1330: PRJNA961726) (Wang et al. 2020; Vela et al. 2024). Mesocotyls of the sorghum cultivar BTx623, inoculated with *C. sublineola*, were used to construct an RNA‐seq database as described previously (Wang et al. 2020), and transcription factors (Supporting Information Table S1) exhibiting more than a 2.0‐fold increase in expression at 48 h post‐infection (compared to the control) were selected to display FPKM values. Additionally, leaf tissue samples from Sc1330 inoculated with *C. sublineola* were used to generate another RNA‐seq dataset as described previously (Vela et al. 2024), with FPKM values shown at 3 d post‐infection.

For phylogenetic and sequence alignment analyses, homologues of SbERF096 and SbWRKY62 were retrieved from NCBI (SbERF096, identity > 50%, query cover > 60%, SbWRKY62, identity > 40%, query cover > 60%) and protein sequences. Sequence alignment was conducted by ClustalW, and a maximum‐likelihood tree was constructed with IQ‐TREE (2.4.0) (Minh et al. 2020), supported by 1000 bootstrap replicates. Best‐fit evolutionary model was LG + I + G for both ERFs and WRKYs. For phylogenetic analysis of XIPs (retrieved from Phytozome V14), sequence alignment was conducted by ClustalW, and a maximum‐likelihood tree was constructed with MEGA software, supported by 1000 bootstrap replicates.

### 4.3 Dual Luciferase Reporter (DLR) Assay

Dual luciferase assay is widely used to study the regulatory roles of transcription factors on target promoters. The coding sequence (CDS) regions of 10 MYBs, 12 WRKYs, 4 Zinc Fingers, 1 ERF, 3 bHLHs, 5 NACs (Figure 1) were amplified from infected mesocotyls cDNA and cloned into pGreenII‐62‐SK vectors as the effectors. The promoter sequences of *SbSTS1*, *SbSOMT* and *SbXIP2*, *SbLRR1*, *SbXIP1*, *SbHSP70*, *SbGT1*, *SbPR5K*, *Sb06g000810*, *SbCYP76M5* and *SbPME31* were amplified and recombined to pGreenII‐0800‐LUC vectors as reporters. Accession numbers of these target genes were listed in Supporting Information Table S2. All constructs were transformed into *Agrobacterium tumefaciens* strain GV3101 with *pSoup* as a helper plasmid.

Transient expression assays were performed in 4–6‐week‐old tobacco (*N. benthamiana*) leaves. Mixture of *Agrobacterium* strains harbouring effector and reporter genes, at a ratio of 10:1, were resuspended in infiltration buffer containing 10 mM MgCl_2_, 10 mM MES, and 100 μM acetosyringone, adjusted to an OD600 of 0.8, and infiltrated into tobacco leaves. For each biological replicate, the fifth fully expanded leaf of each plant was infiltrated. Each biological replicate represented one individual plant, with six biological replicates included for each assay. After 3 days of incubation, the enzymatic activities of Firefly luciferase and *Renilla* luciferase were measured by Dual Luciferase Reporter Assay Kit (Vazyme, DL101‐01). A total of 6 biological replicates were carried out for each experiment. Relative luciferase activity was calculated as the Firefly/Renilla ratio and normalised to the corresponding empty‐vector control. For the DLR assay shown in Figure 6a, the previously validated p35S::SbWRKY62 and pSbSOMT::LUC combination was included as an internal positive control. For assays with multiple transcription factors tested on the same promoter, differences were analysed using one‐way ANOVA followed by Dunnett's multiple comparison test. For assays with different promoters tested with the same transcription factor, each promoter was analysed separately using an unpaired two‐tailed Student's t‐test. Statistical significance was denoted as *p < 0.05 and ***p < 0.001.

### 4.4 Protoplasts Isolation and Transformation

Sorghum cultivar DK18 (Lo and Nicholson 1998) was used as the source for protoplast isolation performed as described previously with minor revisions (Yoo et al. 2007; Meng et al. 2020). Prior to planting on MS agar medium, seeds were surface‐sterilised with 50% bleach (25 min) and 70% ethanol (5 min), followed by three sterile water rinses. Plants were grown under a 12 h day/night photoperiod at 29°C (day) and 20°C (night). Protoplasts were isolated from the mid‐region of 18‐day‐old leaf blades which were cut into 0.2 mm strips. Following a 10‐min pretreatment in 0.5 M mannitol in the dark, tissues were digested in enzyme solution (20 mM MES, PH5.8, 1 M mannitol, 1% (wt/vol) Cellulase, 0.25% (wt/vol) Macerozyme, 10 mM CaCl_2_) for 4 h at 40 rpm and then 1 h at 80 rpm. After adding an equal volume of W5 solution (154 mM NaCl, 2 mM MES, pH 5.8, 5 mM KCl, 125 mM CaCl_2_), protoplasts were collected by centrifugation at 110×g, resuspended, and transfected with different constructs via the polyethylene glycol‐calcium method (Yoo et al. 2007). Sorghum protoplasts transformed with the empty vector were used as the negative control (NC).

### 4.5 RNA Isolation and qRT‐PCR Analysis of Gene Expression

Total RNA was isolated from sorghum mesocotyls and protoplasts by FreeZol Reagent (Vazyme, R711). qRT‐PCR experiments were performed using PrimeScript RT reagent kit with gDNA eraser (TaKaRa, Japan) and TB Green premix Ex Taq II kit (TaKaRa, Japan), respectively. The qRT‐PCR cycling conditions were as follows: 95°C for 30 s, followed by 40 cycles of 95°C for 5 s and 60°C for 30 s. Primers used in qRT‐PCR are listed in Supporting Information Table S3. The sorghum housekeeping gene *Eukaryotic Initiation Factor 4A‐1* (*SbEIF4α*, XM_002451491) was used as an internal control. Relative expression levels were calculated using the 2^‐ΔΔCt^ method (Livak and Schmittgen 2001). The empty‐vector control and the 0 h sample were used as calibrators for the protoplast transactivation assays and pathogen‐inoculation experiments, respectively. Three independent biological replicates were used for each sample.

### 4.6 LC‐MS/MS Metabolite Analysis of *Agrobacterium*‐Infiltrated Tobacco Leaves

CDS regions of SbSTS1 and SbSOMT, isolated from cDNA of infected SC748‐5 mesocotyls, were cloned into pGreenII‐0800‐SbSTS1‐pro‐luc and pGreenII‐0800‐SbSOMT‐pro‐luc vectors, respectively, by replacing the luciferase CDS. The resulting constructs were designated pGreenII‐0800‐SbSTS1‐pro‐SbSTS1 and pGreenII‐0800‐SbSOMT‐pro‐SbSOMT. They were transformed into *Agrobacterium tumefaciens* strain GV3101 with *pSoup* as a helper plasmid.

Two *Agrobacterium* cultures were prepared: one harbouring the effector constructs in the pGreenII‐62‐SK vector, including SbERF096, SbWRKY62, Sb01g040280 or the empty SK control, and the other harbouring the modified reporter vectors. When the optical density of each culture reached 0.8 at 600 nm, they were mixed in a 1:1 ratio and co‐infiltrated into tobacco leaves. For Figure 3b, Sb01g040280, which did not activate the *SbSTS1* promoter in the DLR assay, was included as a non‐activating ERF negative control. For co‐expression assays, Agrobacterium cultures carrying the indicated constructs were adjusted to the same OD600 value, mixed before infiltration, and co‐infiltrated into the same tobacco leaf area. Metabolites were extracted after 5 days of incubation as described previously (Lui et al. 2023). Briefly, 100 mg tobacco leaves were frozen in liquid nitrogen, and ground to fine powder using TissueLyser II (QIAGEN, Germany). Each sample was ultra‐sonicated in 200 μl 80% (vol/vol) methanol on an ice‐water bath for 30 min and filtered through a 0.22 μm PTFE membrane filter (Phenomenex, USA) prior to LC‐MS/MS analysis. A total of 3 biological replicates were used.

For the detection of piceid and pterostilbene, HPLC‐Q‐AP3200‐QTRAP‐MS‐MS via multiple reaction monitoring (MRM) were used. Metabolites were separated by Kinetex C18 column (Kinetex 2.6 μm C18 100 Å, 100 × 2.1 mm, Phenomenex, USA) with the linear gradient 10%–90% B under a flow rate of 0.5 ml min^−1^. The mobile phases were 0.5% formic acid/water (vol/vol) (A) and 0.5% formic acid/methanol (B). Authentic standards of piceid, also known as polydatin, and pterostilbene were used to confirm retention times and MRM transitions, and external calibration curves generated with these standards were used for quantification. The piceid/polydatin standard was purchased from Sigma‐Aldrich (Cat. No. PHL89312‐20MG), and the pterostilbene standard was purchased from Sigma‐Aldrich (Cat. No. P1499‐10MG).

### 4.7 Yeast One Hybrid (Y1H) Assay

The promoter sequence of *SbSTS1* (2000bp) was amplified from gDNA of sorghum cultivar SC748‐5 and cloned into pLacZi (Clontech, Kyoto, Japan), and the full length CDS of SbERF096 isolated from SC748‐5 was constructed into pJG45 (Clontech). The MatchmakerTM Gold Yeast One Hybrid Library Screening System (Clontech, Kyoto, Japan) was used to test the interaction. Briefly, two constructs pJG45‐SbERF096 and pLacZi‐*SbSTS1*‐pro were co‐transformed into yeast strain EGY48 and selection medium was used to confirm the interaction. Yeast cells co‐transformed with AD‐T7‐T (T7) and BD‐T7‐53 (53) served as the positive control and Empty pJG45 vector with pLacZi‐*SbSTS1*‐pro was used as negative control as described previously (Ma et al. 2025).

### 4.8 Electrophoretic Mobility Shift Assay (EMSA)

SbERF096 and SbWRKY62 were cloned into pMAL‐c6T vector (NEB, USA, N8180) and transformed into *Escherichia coli* BL21(DE3). The transformed cells were induced by 0.5 mM isopropyl b‐D‐1‐thiogalactopyranoside followed by incubation at 16°C for 24 h. MBP‐tagged protein was purified by Amylose Resin (NEB, USA, E8021S), and eluted by MBP Column Binding Buffer (20 mM Tris‐HCl, 200 mM NaCl, 1 mM EDTA, pH 7.5) containing 10 mM maltose. EMSA was performed using the LightShift™ EMSA Optimisation and Control Kit (Thermo Scientific, Cat. No. 20148X) according to the manufacturer's instructions. For each binding reaction, 0.1 μg of purified MBP‐tagged SbERF096 or SbWRKY62 protein was incubated with 5 nM biotin‐labelled probe in binding buffer. For competition assays, unlabelled competitor probes were added at 50‐, 250‐ and 500‐fold molar excesses.

### 4.9 Chromatin Immunoprecipitation Assay (ChIP)

Chromatin immunoprecipitation (ChIP) assays in sorghum protoplasts were performed as described previously (Tu et al. 2020). SbERF096‐Flag and SbWRKY62‐Flag fusion proteins were over‐expressed in sorghum protoplasts and crossed‐linked with genomic DNA in 4% Formaldehyde (vol/vol). Chromatin was fragmented with a Bioruptor Pico Sonication System (Diagenode, Seraing, Belgium) and immunoprecipitated with an anti‐Flag antibody (Thermo) incubated with protein A or G Magnetic beads (Surebeads, Bio‐rad). DNA fragments were purified by Phenol:Chloroform:Isoamyl alcohol (BioUltra, Molecular Biology, 25:24:1). Real time qPCR was used to quantify the relative enrichment of each fragment as described previously (Susila et al. 2021). All primers used in ChIP assay are listed in Supporting Information Table S4.

### 4.10 DNA Affinity Purification Sequencing (DAP‐seq)

DAP‐seq experiments were conducted by Biorun Biotechnology Co. Ltd. (Wuhan, China) with two biological replicates. The full‐length CDS of SbWRKY62 was cloned into a pHalo‐tag vector for protein expression using a wheat germ system. Genomic DNA, isolated from sorghum SC748‐5 seedlings, was used as the binding library. Raw DAP‐seq reads were trimmed using Trimmomatic v0.39, retaining reads of at least 20 bp, and read quality was assessed using FastQC v0.11.5 with default parameters. Clean reads were aligned to the sorghum reference genome using BWA v0.7.17‐r1188 with default parameters. DAP‐seq peaks were called independently for each replicate using MACS2 v2.2.7.1 with a q‐value cutoff of 0.05. To reduce false positives, only peaks detected in both independent biological replicates were retained. Target genes were assigned when the retained peaks were located within promoter regions, defined as 2 kb upstream of the transcription start site. Genes associated with peaks showing more than 4.0‐fold enrichment were used for KEGG pathway analysis using TBtools (Chen et al. 2023), while a more stringent fold‐enrichment threshold of more than 15 was applied for further shortlisting of high‐confidence candidate target genes. P‐values from KEGG enrichment analysis were adjusted for multiple testing using the Benjamini–Hochberg FDR method.

## Supporting information


Supporting File 1

## Data Availability

The data that support the findings of this study are available from the corresponding author upon reasonable request.
