# Supplementary material for: SbWRKY62 Coordinates With SbERF096 to Activate Pterostilbene Biosynthesis and Targets a Xylanase Inhibitor Protein for Anthracnose Resistance in Sorghum
Source: Plant Cell Environ. 2026 Aug 19;49(12):9524–39. doi: 10.1111/pce.70833 (PMC13725255; doi:10.1111/pce.70833)
Supplement: Supplementary file 1 [file PCE-49-9524-s001.docx]

Fig. S1 Screening and identification of MYBs, WRKYs, and TFs from other families. (a) Schematic diagram of stilbene biosynthesis in sorghum. (b) Promoter analyses of *SbSTS1* and *SbSOMT*. MYB, ERF, bZIP, HD-ZIP, bHLH, Zinc Finger, NAC and WRKY binding sites exist in promoters of both *SbSTS1* and *SbSOMT*. (c) Distribution of differentially expressed genes (DEGs) and transcription factor (TF) genes among the candidate genes.

Fig. S2 FPKM of MYBs, WRKYs, Zinc fingers, ERF, bHLHs and NACs in RNA-seq database (BTX623, PRJNA525011, SC1330, PRJNA961726). Sorghum cultivars, BTX623 and SC1330, infected by *C.sublineola*.

Fig. S3 Expression pattern of *SbERF096*, *SbWRKY62* after infection by *C.sublineola* in susceptible cultivar, BTx623 and resistant cultivar, SC748-5. Expression values were calculated using the 2^-ΔΔCt method, with the 0 h sample serving as the calibrator. Values refer to means ± SE (n=3).

Fig. S4 Phylogenetic analysis of SbERF096 and homologs in other species. Clade I, homologs in non-Poaceae monocots and dicots. Clade II, homologs in Zizania. Clade III, homologs in Pooideae. Clade IV, homologs mainly in Panicoideae. Clade V, homologs in Phragmites and Chasmanthium. Bootstrap support values are indicated by circles at the nodes, and only values ≥80% are shown. SbERF096 is highlighted in red. Colored backgrounds indicate different clades.

Fig. S5 Phylogenetic analysis of SbWRKY62 and homologs in other species. Clade I, homologs in Paniceae. Clade II, homologs in andropogoneae. Clade III, homologs in Phragmites and Chasmanthium. Clade IV, homologs in Chloridoideae. Clade V, homologs in Pooideae. Clade VI, homologs in Oryzoideae. Clade VII, homologs in Aristidoideae. Bootstrap support values are indicated by circles at the nodes, and only values ≥80% are shown. SbWRKY62 is highlighted in red. Colored backgrounds indicate different clades.

Fig. S6 Predicted binding sites of 9 target genes recognized by SbWRKY62 in DAP-seq. Light coral pink peaks indicated 2 replicates of SbWRKY62 DAP-seq and blue peaks indicated Input.

Fig. S7 Expression pattern and phylogenetic tree of SbXIP1. (a) Expression pattern of *XIPs* in sorghum after infection by *C.sublineola* and control. Values refer to means ± SE (n=3). (b) Phylogenetic tree of XIPs in sorghum and XIPs reported in other monocots. The XIP protein sequences from *Sorghum bicolor* were depicted in powder blue, whereas SbXIP1 was denoted in light coral pink. (c) qRT-PCR analysis of *SbXIP1* expression in BTx623 and SC748-5 at 24, 48, and 72 h after *C. sublineola* infection. Values refer to means ± SE (n=3).

Table S1 Accession numbers of TFs

|  | Accession number |
| --- | --- |
| SbJAMYB | Sb08g018370 |
| SbJAMYB-like | Sb05g026820 |
| SbM000480 | Sb05g000480 |
| SbMYB20-like | Sb10g009330 |
| SbMYB2 | Sb02g043420 |
| SbMYB2-like | Sb09g002675 |
| SbMYB117 | Sb03g010970 |
| SbMYB4 | Sb04g026480 |
| SbMYBPHL11 | Sb06g027405 |
| SbMYBODORANT1 | Sb02g024250 |
| SbWRKY6 | Sb03g000240 |
| SbWRKY28 | Sb10g025600 |
| SbWRKY58 | Sb01g007480 |
| SbWRKY28-like | Sb10g025590 |
| SbWRKY65 | Sb03g033640 |
| SbWRKY75 | Sb05g017130 |
| SbWRKY71-like | Sb03g028530 |
| SbWRKY72 | Sb03g003370 |
| SbWRKY24 | Sb09g023270 |
| SbWRKY13-like | Sb03g032800 |
| SbWRKY53 | Sb09g015900 |
| SbZAT12 | Sb01g039190 |
| SbZAT36 | Sb01g031920 |
| SbZAT10 | Sb01g031900 |
| SbZinc Finger 8 | Sb02g032630 |
| SbbHLH041 | Sb03g002910 |
| SbbHLH51 | Sb03g005250 |
| SbbHLH148 | Sb09g006220 |
| SbJUNGBRUNNEN 1 | Sb01g006410 |
| SbNAC29 | Sb05g001580 |
| SbNAC102 | Sb02g043210 |
| SbNAC2 | Sb05g003400 |
| SbNAC1 | Sb10g027100 |

Table S2 Accession number of target genes from DAP-seq of SbWRKY62

|  | Accession number |
| --- | --- |
| SbXIP2 | Sb05g023710 |
| SbLRR1 | Sb03g027080 |
| Sb06g000810 | Sb06g000810 |
| SbHSP70 | Sb02g028570 |
| SbGT1 | Sb03g034410 |
| SbPR5K | Sb03g008170 |
| SbCYP76M5 | Sb02g007420 |
| SbPME31 | Sb01g022290 |
| SbXIP1 | Sb05g027380 |

Table S3 Primers used in qRT-PCR

| Gene | Primers in qRT-PCR |
| --- | --- |
| SbSTS1_F | TGCTACGGTGTTGGCCATT |
| SbSTS1_R | ATCGACTTGTGGCATATCCTCTT |
| SbSOMT_F | AGGTGGCAGCATGTTCGATAA |
| SbSOMT_R | ACGATCACCTTGCCTCTCAC |
| SbWRKY6_F | AGTGGCGCAAGTACGGGCAGAA |
| SbWRKY6_R | GGACGAAGACTGGTGGGCGTTGTA |
| SbWRKY62_F | TCACTTGAAACACCTGTGCCG |
| SbWRKY62_R | GGTGCTTGATATGCTGGAGTGG |
| SbJAMYB _F | TGAAGCGGACGGGCAAGAGC |
| SbJAMYB _R | TGCGGCATCCAGAGGTAGCG |
| SbM000480_F | CTCCACCTCTGCTTCCTCCG |
| SbM000480_R | CCTGGTCCGAGTCGTCGAT |
| SbMYB2-like _F | CCGCCCGCGTACTGTTATG |
| SbMYB2-like _R | GCAGCCTTGGTCGTTCGTC |
| SbERF096_F | GTGAAGGGAGAGGGGAATGAAATG |
| SbERF096_R | ATACTACTCTGCCTCTGCCTCT |
| SbEIF4α_F (Housekeeping gene) | CAACTTTGTCACCCGCGATGA |
| SbEIF4α_R (Housekeeping gene) | TCCAGAAACCTTAGCAGCCCA |
| SbXIP2_F | ATGTGTGAGTGTGTGACTAC |
| SbXIP2_R | TGTAGGGTACTTACGGACTAC |
| SbLRR1_F | CACCGTTCAATCCATGAAAC |
| SbLRR1_R | AGACCGACATACTTCAGCT |
| Sb06g000810_F | TACGCTATCCGCCACTCGATC |
| Sb06g000810_R | TCGAAGACCGGGTAGCTGAAG |
| SbHSP70_F | GTACAAGGACAAGGTACCTGCTG |
| SbHSP70_R | CTAAGAAGCCGGAGGTAGGTAAG |
| SbGT1_F | AGCTCCGAAGTCACACTCACA |
| SbGT1_R | AGGCGGTTGACTTGACGGG |
| SbPR5K _F | ACTATGGAATCACCATGGCCACC |
| SbPR5K R | TGCACCACATGATAGAGGGCT |
| SbCYP76M5_F | AGAGTAGTCAGATAGCAGATGCACAC |
| SbCYP76M5_R | GTTGCCGATGAGCGGCAGA |
| SbPME31_F | ATGTTGAAGCAGAGCAGTTCC |
| SbPME31_R | ATTAGATGGGCTACAAAGGAGGC |
| SbXIP1_F | GTCAAGGAGGCGCTCGACA |
| SbXIP1_R | ATGCTGCCGCTGTAGTTGC |

Table S4 Primers used in CHIP-qPCR

| Gene promoter | Primers in ChIP-qPCR |
| --- | --- |
| SbSTS1_GCC_F | ATGAAACCAAGCGGCCCTAAC |
| SbSTS1_GCC_R | CTGCAGAGGTTGCACATCTTTAC |
| SbSTS1_RAA_F | GACCATGATCCTGTGACTAGAT |
| SbSTS1_RAA_R | GTAACCGCTAACGAGCTAG |
| SbSOMT_S1_F | TCCCTCCGGTCCTATATATCTGGC |
| SbSOMT_S1_R | CGGAGTGGGGAGACCG |
| SbSOMT_S2_F | GGCACATGAATTATTAGTCCATGGTCA |
| SbSOMT_S2_R | GCTATCAGACCGTACGTCCCGA |
| SbSOMT_S3_F | ACGGTCTGATAGCTGCGT |
| SbSOMT_S3_R | AACTGTTCTAGCTACATAGGAGCTT |
| SbXIP1_WT1_F | CCAACGCTCGACGAGATG |
| SbXIP1_WT1_R | AGCCTTCTCTACAGTTCGGTCG |
| SbXIP1_WT2_F | CGACCGAACTGTAGAGAAGGCT |
| SbXIP1_WT2_R | CTTGAAAGAGACATGAGTGCAACG |
| SbXIP1_WT3_F | GACGAGAACTAAACAAGACCAGCTG |
| SbXIP1_WT3_R | ATATGGAGAGACCGAATCGGAAAGAC |
| SbXIP1_NC_F | TGTGGACGACAAGATTTGCAATG |
| SbXIP1_NC_R | CAATATGGATCATAAGATTCGCAATGAG |
